# The Need of Dental Instruction in Medical Schools

**Published:** 1898-06

**Authors:** Edward Branigan

**Affiliations:** Boston, Mass.


### The Need of Dental Instruction in Medical Schools.

BY EDWARD BRANIGAN, D.D.S., BOSTON, MASS.

Presented to the Section on Stomatology, at the Forty-eighth Annual Meeting of the American Medical Association, held at Philadelphia, Pa., June 1-4, 1897.

There is a great need of dental instruction in medical schools. Physicians should know more about the teeth than they now do, and when medical students are given a better knowledge of the teeth and of the many disturbances they cause, then will medical science do more for humanity than it is now doing. More than a dozen years spent in looking into the mouths of my neighbors, at the rate of hundreds of mouths a week, has revealed to my observation so many instances of improper treatment of diseases caused by the teeth, that I have long felt it my duty to protest, and I now take advantage of the opportunity given me to put my protest into the relation of experiences that have come to me so many times that I put them forth as cold facts. I shall confine myself to the narration of a few of the more common instances of mismanaged treatment of diseases caused by the teeth. The evils I speak of are common.

Abscesses caused by pulpless teeth are still poulticed and opened on the face by medical men. I have seen many hundreds of these, for this treatment never effects a cure, and in the course of time it dawns upon the patient, if not upon the doctor, that dental assistance is needed. How much better it would have been for the patient to have been sent to the dentist at first and so avoided days and weeks of misery and the necessity of carrying a hideous scar for life. People are still being stabbed on the face and neck because a third molar sometimes causes trouble in trying to get up into the mouth where it belongs. The medical treatment for these simple abscesses is sometimes varied a little. The sufferer may be kept in bed for a time, with temperature and pulse watched, and medicine changed at intervals, according to the symptoms. This sometimes happens when the old tooth-root, at the bottom of the trouble, is so loose that the owner could remove it with two fingers. Pus from an abscessed tooth is frequently discharged into the antrum. The extraction of the tooth, followed by proper treatment, would rob the medical profession of many patients who are supposed to suffer from, and are treated for, catarrh. Serious throat diseases are frequently caused by pulpless teeth and sometimes by unerupted or retained teeth. All throat specialists are not aware of this fact.

A more intimate knowledge of the mouth and teeth would help other specialists, aurists, for instance. Severe and painful inflammations of the ear, caused by teeth, are common. The removing of fillings from pulpless teeth has made many sufferers from improper aural treatment happy. Unerupted wisdom and occasionally unerupted supernumerary teeth are the cause of many obscure ear troubles. Next to the dentist, no one needs dental knowledge more than the aurist. Oculists are now tracing a variety of diseases of the eye back to the teeth, but they have not yet found out all the mean things a diseased tooth can do. Many sufferers from irritation of the eye, backed up by an inflamed tooth, do not get to the oculist, and if the general practitioner knew more about the teeth as a cause of disease, he would not in so many of these cases prescribe an eye-wash, or glasses, or a tonic; he would send the patient to a dentist.

A tumor of the nose may be caused by a dead tooth-pulp. Treatment of the tumor without a knowledge of the cause is not calculated to make the owner of the nose happy. The victims of so-called neuralgia, who after weeks and months and even years of anti-neuralgic drugging, die or find relief at the hands of some one who knows something about teeth, are countless. Thousands of people are today being drugged for neuralgia who are really suffering from some form of dental irritation. A pulp-stone no larger than a pin-head can bring about so much pain and loss of sleep, appetite and health that nervous prostration may be, and often is, the end of an affair that a pair of forceps would have ended in a moment.

Bicycle riding has given relief to many sufferers from dyspepsia, but many more dyspeptics would get better if their medical advisers insisted upon their having all their lame and tender teeth cared for. Among the hundred or two of mouths opened every morning for my inspection, I never fail to find a number showing evidence of being used on one side only, and in many of the mouths the front teeth are the only ones that do any work.

Physicians should also take into consideration the constant presence of pus in the mouths of many of their patients. They do not all do this. I find pus in physician's mouths about as often as I find it in the mouths of other classes of patients.

The authorities in charge of the management of hospitals should know more of dentistry. If they did, the nurses and other hospital attendants would know how to cleanse a patient's mouth, and the hospital surgeon would know how to look into a patient's mouth intelligently. The removal of half or a whole jaw for necrosis, would not take place when the extraction of a few roots and the use of an antiseptic mouth-wash would effect a cure. When hospital surgeons know more about the mouth and jaws we shall not be obliged to meet people who have been dismissed from hospitals after some weeks of treatment for broken jaw, with the fracture ununited or with the union in so erratic a form that the sufferer can not close his jaws. When some one about the hospital is able to examine a mouth we shall not be called on to help the man who takes his broken jaw away from the hospital after having been assured that he will be all right in a few days if he puts a poultice on his bruised face and takes a few pills. It is a fact that some surgeons can not locate a fracture of the jaw, and it is also a fact that when located, fractured jaws are sometimes treated by being bound together with a four-tailed bandage, and then, as a happy afterthought, a couple of front teeth are pulled out to make a hole through which to pass the patient's nourishment.

After dental instruction is given to medical students, the army and the navy will get a little much needed dental service. They get very little now, although hospital stewards are supposed to be able to pull teeth.

The medical directors of public institutions will find a vast amount of good work ready for them when they realize the importance of having dental service given to those in their charge. A few years ago I discovered that not one of the 500 children in one of the public institutions in my city had a tooth-brush. The children of that institution are now supplied with tooth-brushes, but I fear there are many similar institutions in the world where the tooth-brush is never seen and the dentist never heard of. I am sure that this evil will be corrected very soon after dental instruction is given to the future medical directors of public institutions for the care of the poor.

